# Patient-Specific Finite Element Analysis of Ventral Incisional Hernia Repair: Biomechanical Investigation of the Cause of Recurrence

**DOI:** 10.3390/bioengineering13070801

**Published:** 2026-07-13

**Authors:** Tochukwu Bright Ezechukwu, Yigang Luo, Sadman Sakib, Akinola Ogbeyemi, Jethro Odeyemi, Nara Song, Wenjun Zhang

**Affiliations:** 1 Division of Biomedical Engineering, College of Engineering, University of Saskatchewan, Saskatoon, SK S7N 5A9, Canadaxns319@usask.ca (S.S.); akinola.ogbeyemi@usask.ca (A.O.); jethro.odeyemi@usask.ca (J.O.); 2 Division of General Surgery, College of Medicine, University of Saskatchewan, Saskatoon, SK S7N 5A9, Canada; 3 Biomedical Sciences, College of Medicine, University of Saskatchewan, Saskatoon, SK S7N 5A9, Canada; vyb871@mail.usask.ca; 4 School of Mechanical Engineering, Donghua University, Shanghai 200051, China

**Keywords:** finite element method, stress, suture, tissue biomechanics, ventral incisional hernia

## Abstract

Ventral incisional hernia is a frequent postoperative complication following abdominal surgery, with recurrence largely driven by biomechanical factors such as elevated intra-abdominal pressure and stress concentration at the suture–mesh–tissue interface. This study presents a patient-specific finite element analysis (FEA) framework to investigate stress transfer mechanisms within ventral incisional hernia repair systems under physiologically relevant loading conditions. A patient-specific abdominal wall geometry was reconstructed from computed tomography images and modeled as a nonlinear hyper-elastic material using a second-order Ogden formulation. Polypropylene surgical meshes and sutures were represented using finite elements, and an intra-lay mesh placement with a midline incision was simulated. Loading conditions corresponding to regular breathing, forceful breathing, and heavy-load lifting were applied. The results reveal that stress concentrations consistently localize at the sutures, with stress magnitudes increasing markedly under higher physiological loads. Under heavy lifting, suture stresses approached the material yield limit, whereas mesh stresses remained comparatively low. These findings identify suture failure as a critical mechanical factor contributing to hernia recurrence and highlight the importance of postoperative load management and improved support strategies. This work provides a basis for more comprehensive future patient-specific analyses.

## 1. Introduction

The abdominal wall serves as a critical structural and functional barrier, protecting the intra-abdominal organs from mechanical injury and maintaining abdominal integrity [1]. Following surgical interventions, the muscular and fascial layers of the abdominal wall may undergo weakening or disruption, leading to a reduction in tensile strength [2]. This compromised structural integrity can result in the formation of a defect through which intra-abdominal tissues or organs may protrude, giving rise to a ventral hernia. Clinically, a ventral hernia is characterized by the protrusion of abdominal contents through a weakened or defective region of the abdominal musculature or fascial layers [3].

Ventral incisional hernia is a common postoperative complication, with reported incidence rates ranging from approximately 3% to 20% among patients undergoing abdominal surgical repair [4,5]. Despite advances in surgical techniques, recurrence of incisional hernia remains a persistent clinical challenge and is widely recognized as a multifactorial phenomenon influenced by patient-related, surgical, and biomechanical factors [6]. Over several decades, extensive efforts have been made to reduce hernia recurrence following abdominal surgery through a variety of repair strategies, including procedures performed with or without prosthetic mesh reinforcement.

The two most widely adopted surgical approaches for incisional hernia repair are open surgical repair and laparoscopic repair [7]. In an open surgical repair, an incision is made directly over the hernia defect, and a prosthetic mesh is sutured to reinforce the weakened abdominal wall. This approach is commonly employed for both small and large hernias, particularly in patients with a history of previous hernia repairs [8]. In contrast, laparoscopic repair involves the insertion of small cannulas (laparoscopic ports) through the abdominal wall, allowing the surgeon to access and repair the defect using minimally invasive instruments [9]. During a laparoscopic repair, the hernia defect is closed and, depending on the clinical indication, a mesh may be placed to provide additional reinforcement.

Despite the clinical success of both open and laparoscopic techniques, recurrence remains a significant concern. Following surgery, patients are exposed to various physiological loading conditions during routine activities such as lifting, coughing, sneezing, and strenuous use of thbe abdominal muscles [10]. These activities generate elevated intra-abdominal pressures and mechanical stresses on sutures and implanted meshes. Over time, repeated loading can lead to suture failure, mesh displacement, or loss of fixation, thereby compromising the integrity of the repair and increasing the likelihood of hernia recurrence. In many cases, excessive stress concentration at the suture–mesh–tissue interface results in tearing of sutures or gradual migration of the mesh from its original position, ultimately leading to reherniation.

The motivation of the present study is to advance technological and biomechanical understanding aimed at reducing the recurrence of ventral incisional hernia following surgical repair. To address this challenge, this work develops a mathematical and computational framework capable of simulating stress distributions within the abdominal wall, sutures, and surgical mesh under physiologically relevant loading conditions. The primary objective of this study was to construct a finite element model of the abdominal wall–suture–mesh system to evaluate the mechanical response when intra-abdominal contents exert pressure on the repaired hernia site. Finite element analysis (FEA) [11,12] was employed to characterize stress transfer mechanisms and deformation behavior within the repair construct.

To achieve this objective, established procedures reported in the literature were adopted and applied to the modeling and reconstruction of the abdominal wall. Anatomical data were obtained from medical imaging of a male subject with a mean body mass index (BMI) of less than 25 kg/m^2^ and an age range between 19 and 23 years, processed using the open-source imaging software 3D Slicer. This study contributes to the field of surgical biomechanics by integrating computational modeling, numerical simulation, and mechanical analysis to provide insight into stress-induced failure mechanisms in ventral incisional hernia repair. The findings aim to inform improved surgical strategies and device designs that may reduce recurrence rates and enhance long-term clinical outcomes.

Compared with previous finite element studies of abdominal wall biomechanics, the present work introduces several features that enhance the physiological relevance of the analysis. First, a patient-specific three-dimensional abdominal wall geometry reconstructed from computed tomography images was employed rather than simplified generic geometries. Second, the nonlinear mechanical behavior of the abdominal wall was represented using a second-order Ogden hyperelastic constitutive model, which more accurately captures the deformation characteristics of soft biological tissues. Third, clinically relevant polypropylene mesh and suture configurations were incorporated to simulate the mechanical interactions occurring at the repair site. In addition, loading conditions corresponding to different physiological states were considered to reproduce the mechanical effects associated with daily activities. These features enable a more realistic assessment of stress transfer mechanisms and provide insight into the role of suture stress concentrations as a potential cause of ventral incisional hernia recurrence.

## 2. Methodology

In this study, a step-by-step finite element analysis (FEA) workflow was developed to simulate ventral incisional hernia repair by integrating patient-specific abdominal wall geometry with surgical mesh and suture representations. The methodology includes image-based geometry reconstruction, model preprocessing, material definition, mesh generation, and the application of clinically relevant loading and boundary conditions to evaluate stress transfer within the repair system.

### 2.1. Image Acquisition and Geometry Reconstruction

A comprehensive review of existing finite element-based studies on abdominal wall biomechanics was conducted to guide model development. Anatomical reconstruction of the abdominal wall was performed using computed tomography (CT) imaging data from a male subject with a normal body mass index (BMI < 25 kg/m^2^) and age range between 19 and 23 years. The imaging data was obtained and processed using 3D Slicer (Version: 4.11), an open-source platform widely used for biomedical image analysis [13].

The CT images were acquired using a Siemens SOMATOM Definition Flash (Siemens Healthineers, Forchheim, Germany) with a slice thickness of 1 mm, tube voltage of 120 kVp, tube current of 200 mA, pixel spacing of 0.742 mm × 0.742 mm, and an image resolution of 512 × 512 pixels. These imaging parameters ensured adequate spatial resolution for reconstructing the abdominal wall geometry and associated anatomical structures.

The CT images were segmented to reconstruct the abdominal contents and muscular layers. Both manual and semi-automatic segmentation techniques were employed to ensure accurate delineation of anatomical structures. The resulting three-dimensional (3D) models were exported in STL format for subsequent preprocessing and finite element modeling.

### 2.2. Segmentation of the Abdominal Wall

**Figure 1:**
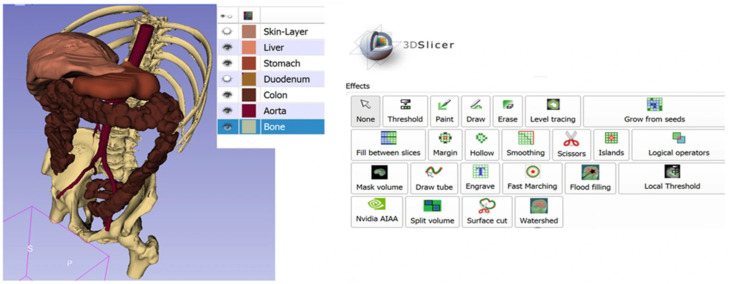
Segmentation and 3D Reconstruction of Abdominal Organs and Skeletal Structures in 3D Slicer.

Segmentation was performed using the Segment Editor module in 3D Slicer, which enables the conversion of 2D medical images into 3D anatomical geometries based on tissue-specific intensity thresholds. The abdominal wall muscles were segmented as regions of interest in both 2D slice views and 3D space. In this paper, the editor tools were used to segment the muscle layer of the abdominal wall as shown in Figure 1.

Although the abdominal wall consists of four distinct muscle groups—rectus abdominis (RA), external oblique (EO), internal oblique (IO), and transversus abdominis (TrA) [14]—these layers were represented as a single equivalent muscle layer in the present model to reduce computational complexity and facilitate convergence of the finite element simulations. Furthermore, the available CT images did not provide sufficient information to accurately distinguish the fiber orientations and anisotropic properties of the individual muscle layers. Similar homogenized representations have been adopted in previous biomechanical studies when patient-specific information regarding layer thickness and fiber architecture is unavailable [15,16]. While this simplification preserves the overall mechanical response of the abdominal wall, it is recognized that neglecting the anisotropic behavior of the individual muscle layers may influence the magnitude and localization of stresses, particularly in regions surrounding the mesh–suture interface where stress concentrations occur. Therefore, the present model is intended to capture the overall stress transfer mechanisms within the repair system rather than predict exact local tissue stresses. Future work will incorporate multilayer anisotropic constitutive models to provide a more physiologically realistic representation of the abdominal wall.

### 2.3. Geometry Cleanup and Preprocessing

**Figure 2:**
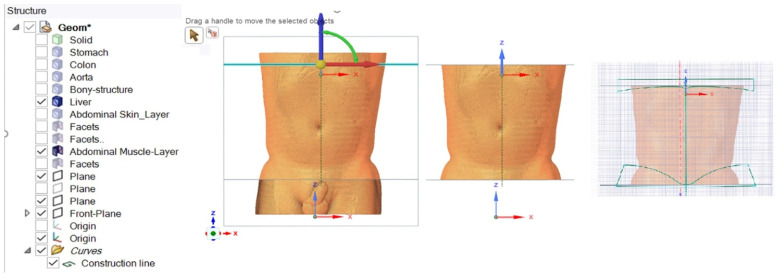
Preprocessing of STL File in ANSYS SpaceClaim.

**Figure 3:**
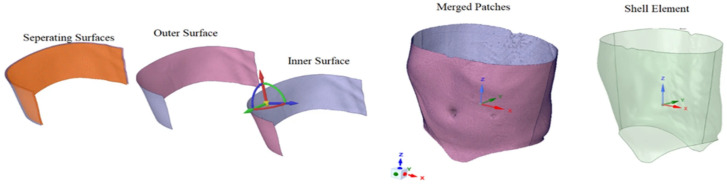
Creating Shell Element (Extracted Surface).

The STL geometry generated from segmentation was imported into ANSYS (Version: 2020 R2) SpaceClaimfor preprocessing (Figure 2). Due to common STL-related issues such as inverted normals, intersecting triangles, and irregular edges, geometry cleanup was performed prior to finite element modeling. The abdominal wall was converted into a shell representation by extracting the inner surface, merging surface patches, and assigning a uniform thickness corresponding to the selected muscle layer thickness (Figure 3). The shell offset was defined toward the bottom surface to represent the physiological orientation of the abdominal wall. This preprocessing step ensured numerical stability and compatibility with shell-based finite element analysis.

### 2.4. Surgical Mesh and Suture Modeling

A synthetic, non-degradable polypropylene (PP) surgical mesh was selected for this study, reflecting its widespread clinical use in ventral incisional hernia repair [17,18]. The mesh was modeled as a woven structure and assumed to exhibit isotropic mechanical behavior [19]. A square mesh geometry measuring 125 mm × 125 mm was generated in SolidWorks 2021 based on dimensions reported in the literature [20]. The mesh porosity was defined to exceed 1 mm to reduce the risk of fibrotic tissue entrapment [21].

**Figure 4:**
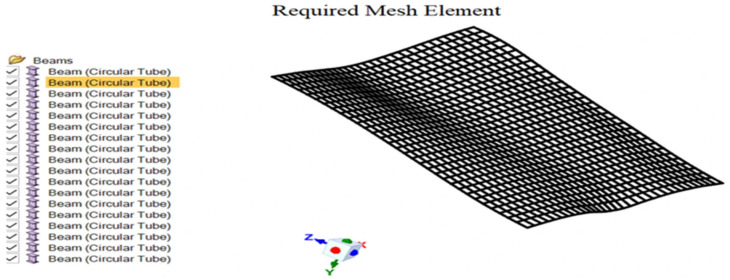
Beam Element for Surgical Mesh.

The mesh geometry was imported into ANSYS (Version: 2020 R2) SpaceClaimand simplified using beam extraction techniques (Figure 4) to reduce computational cost. The mesh fibers were represented as circular beam elements with a diameter of 0.8 mm. Similarly, the sutures were modeled as monofilament, non-absorbable polypropylene materials. The sutures were represented using beam elements with a circular cross-section of 0.7 mm in diameter. Beam representations were selected for both the mesh and the sutures to achieve computational efficiency while preserving mechanical fidelity.

### 2.5. Mesh Placement and Incision Configuration

**Figure 5:**
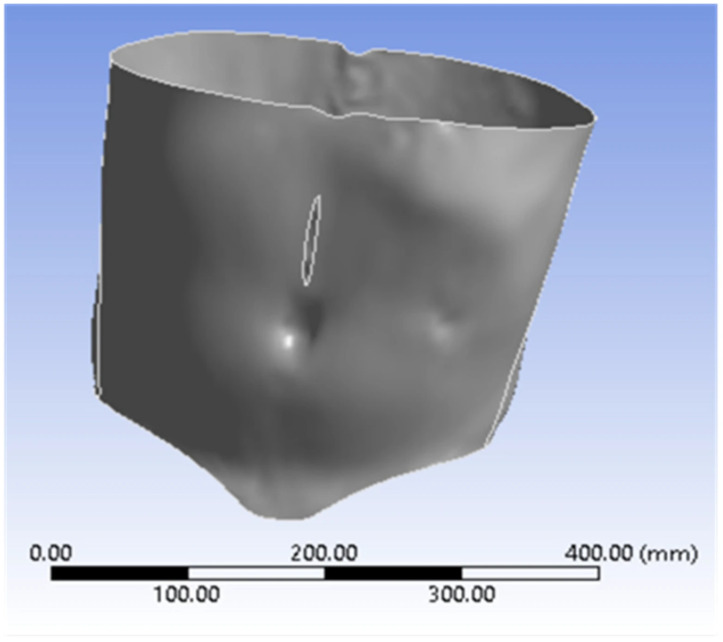
The Midline Incision.

The intra-lay (intraperitoneal onlay mesh, IPOM) placement technique was adopted, wherein the mesh is positioned beneath the abdominal muscle layer and outside the peritoneum. This approach minimizes muscle trauma and is commonly employed in clinical practice. A midline incision configuration was selected, as it is the most frequently used incision type in open abdominal surgeries. Specifically, an upper midline incision was modeled (Figure 5). The finite element model was restricted to the abdominal muscle layer, surgical mesh, and sutures to focus on stress transfer mechanisms at the repair site.

**Figure 6:**
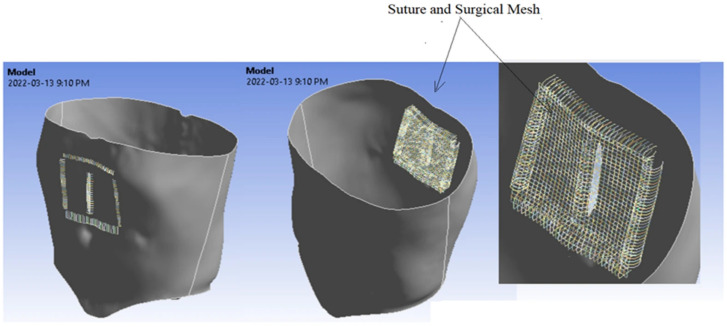
The complete Three-Dimensional Geometry (Beam and Shell Elements).

The complete 3D geometry of the abdominal wall, showing the midline incision, intra-lay placement of the mesh, and sutures, was converted into beam and shell elements in Ansys SpaceClaim, as shown in Figure 6.

### 2.6. Constitutive Model of the Abdominal Muscle

The abdominal muscle layer was modeled as a nonlinear, nearly incompressible hyper-elastic material. A second-order Ogden constitutive model was employed to characterize the stress–strain behavior of the muscle tissue [22]. This model is well suited for biological soft tissues and has been validated in prior biomechanical studies. Specifically, the Ogden material parameters were adopted from the published literature and defined using four constants, μ_1_, μ_2_, α_1_, and α_2_, which were estimated as 0.0439 MPa, −0.236 MPa, 9.82, and −1.00, respectively [23]. These parameters were implemented within the finite element solver to capture the nonlinear deformation response of the abdominal wall under loading.

### 2.7. Finite Element Mesh Generation

**Figure 7:**
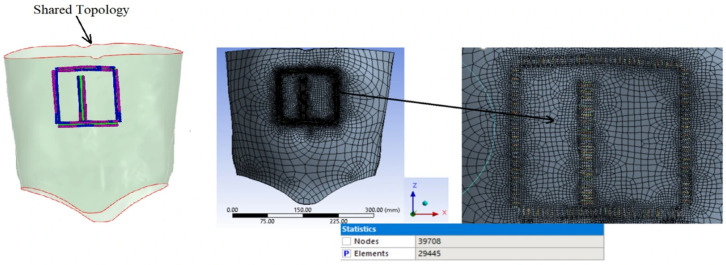
Generated Finite Element Mesh of the Abdominal Wall Model.

Finite element meshing was performed using ANSYS (Version: 2020 R2) Meshing. Geometry defeaturing and virtual topology operations were applied to remove small edges and irrelevant features that could degrade mesh quality. To ensure accurate stress transfer between the sutures and the abdominal wall, topology sharing was implemented at the suture–wall interface (Figure 7). Prior to the final simulations, several global mesh sizes were examined to assess the sensitivity of the numerical results to mesh density. A mesh convergence study was performed by comparing the predicted stress distributions obtained from progressively refined meshes. It was observed that further refinement beyond the selected mesh size resulted in only negligible changes in the calculated stress values while substantially increasing computational cost and solution time. Therefore, a global element size of 60 mm was considered sufficient to provide accurate and numerically stable results. Localized refinement was automatically applied in regions of contact between the mesh, sutures, and the abdominal wall to improve stress resolution in areas of interest. Beam and shell elements were used consistently throughout the model to achieve an appropriate balance between solution accuracy and computational efficiency.

### 2.8. Loading and Boundary Conditions

**Figure 8:**
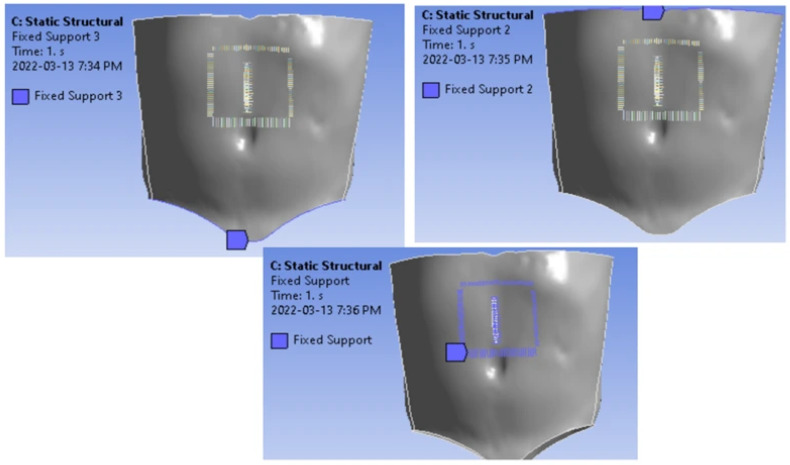
Fixed Supports.

**Figure 9:**
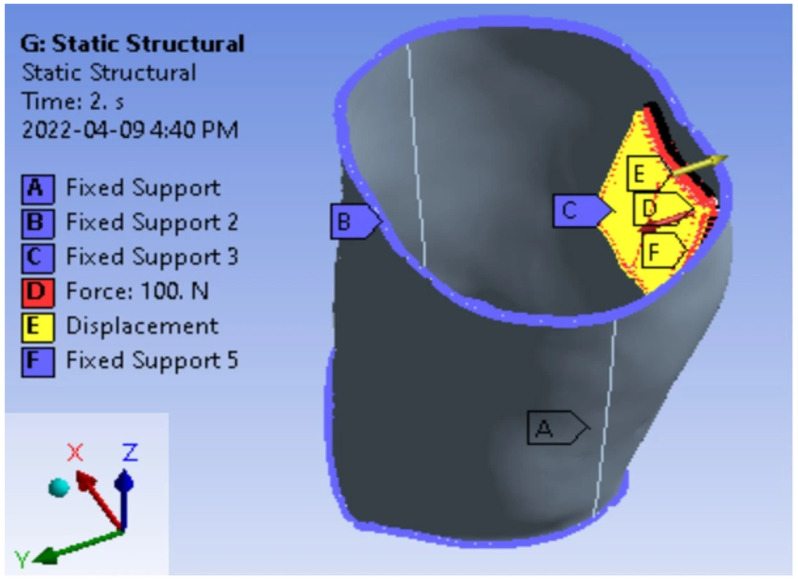
Mesh Displacement and Convergence Plot.

To simulate physiological loading conditions associated with activities such as coughing, deep breathing, and physical exertion, external loads were applied to the repaired abdominal wall. Fixed boundary conditions were assigned to the suture vertices, as well as the top and bottom edges of the abdominal wall, to represent anatomical constraints (Figure 8). Displacement was constrained in the x- and z-directions, allowing motion only along the *y*-axis (Figure 9).

In the present study, physiological loading was represented by a uniformly distributed pressure acting normal to the inner surface of the abdominal wall in the negative y-direction, thereby simulating the mechanical effects of intra-abdominal pressure. The pressure levels were selected based on physiological loading conditions reported in the literature and corresponded to different levels of abdominal activity. The resultant force associated with each loading condition was obtained by integrating the distributed pressure over the loaded area. Three loading states were considered, representing regular breathing, forceful breathing, and heavy-load lifting. These loading conditions corresponded to equivalent resultant forces of 16.05 N, 38.25 N, and 74.3 N, respectively. This loading configuration enabled the evaluation of stress distribution and deformation behavior under clinically relevant conditions and provided a more physiologically realistic representation of the mechanical environment experienced by the abdominal wall–mesh–suture system.

## 3. Results and Discussion

This section presents the finite element simulation results of the abdominal wall–mesh–suture system under physiologically relevant loading conditions and discusses the resulting stress distribution patterns. The findings are interpreted to identify critical mechanical failure mechanisms associated with ventral incisional hernia recurrence and to relate the numerical outcomes to clinical observations.

### 3.1. Physiological Loading Conditions on the Abdominal Wall

An internal report conducted by the College of Medicine at the University of Saskatchewan investigated the magnitude of forces exerted on the abdominal wall under different physiological conditions [24]. The objective of the study was to quantify abdominal wall loading during three expiratory states: regular breathing, forceful breathing, and heavy-load lifting. Participants were tested non-invasively using a dynamometer, and the resulting force ranges are summarized in Table 1. The findings indicated that forces ranging from 11.2 N to 25.1 N were generated during regular breathing, while higher force magnitudes were observed during forceful breathing and heavy-load lifting.

In the present finite element analysis, physiological loading was represented by a uniformly distributed pressure acting over the abdominal surface rather than by concentrated point forces. The resultant force generated by the distributed pressure was used to characterize each physiological state. Based on the average values obtained from the experimental measurements, equivalent resultant forces of 16.05 N, 38.25 N, and 74.3 N were considered for the regular breathing state, forceful breathing state, and heavy-load lifting state, respectively. Thus, the applied loads represent the mechanical effects of intra-abdominal pressure associated with different physiological activities and provide a more realistic description of the loading environment experienced by the abdominal wall–mesh–suture system.

### 3.2. Stress Analysis Framework

Stress develops within a structure when external forces induce internal resistance to deformation. In surgical hernia repair, suturing the mesh to the abdominal muscle layer introduces localized stress concentrations, which may influence the mechanical stability of the repair. To account for the increased stiffness associated with suturing and mesh reinforcement, a stiffness factor of 0.2 was assigned to both the sutures and the surgical mesh in the model. The stress distribution within the model was evaluated using the von Mises stress criterion, which is commonly employed to assess yielding behavior in ductile materials such as polypropylene.

### 3.3. Stress Distribution During the Regular Breathing State (RBS)

During the regular breathing state, an average force of 16.05 N was applied to the internal surface of the abdominal muscle layer, modeled using shell elements. The muscle layer was mechanically connected to the surgical mesh and sutures through mesh connections to enable effective force transfer across the system.

**Figure 10:**
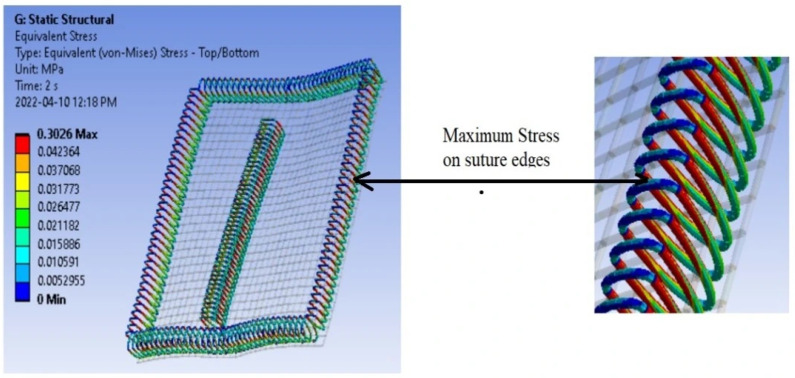
Stress on the suture during the regular breathing state.

**Figure 11:**
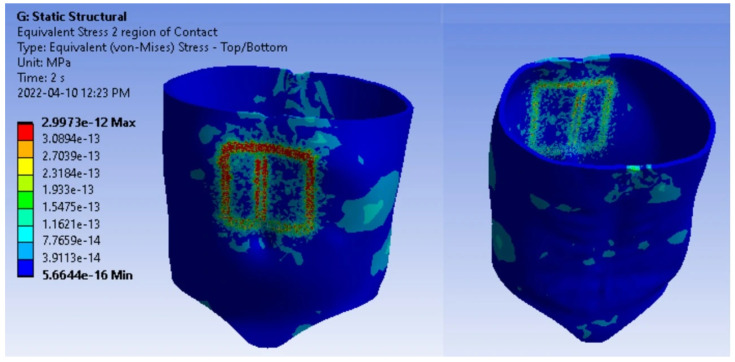
Stress around the areas of contact between the suture and the wall (RBS).

**Figure 12:**
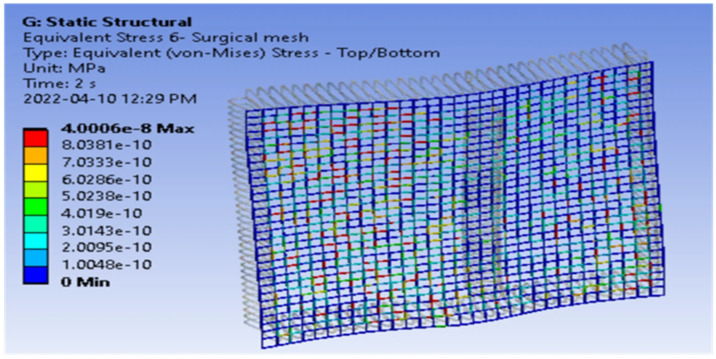
Stress on the surgical mesh during the regular breathing state.

The von Mises stress contours (Figure 10, Figure 11 and Figure 12) revealed that the highest stress concentrations occurred at the suture edges, followed by the contact regions between the sutures and the abdominal wall. These regions of maximum stress were identified by red-shaded areas in the contour plots. Such stress concentrations suggest potential sites of mechanical failure, particularly under repetitive or excessive loading conditions. Elevated stress at the suture–tissue interface may also indicate suboptimal suturing techniques or excessive suture tension.

The nonlinear contact interaction between the suture and the abdominal wall significantly influenced stress distribution. The sutures in the model served to secure the surgical mesh beneath the incision site, and any increase in the applied load led to corresponding increases in localized stress. Failure of the polypropylene sutures is expected if the stress exceeds their yield strength of approximately 2.8 MPa. In comparison, stress observed within the surgical mesh was considerably lower than those in the sutures, indicating that the mesh experienced less mechanical demand during regular breathing.

### 3.4. Stress Distribution During the Forceful Breathing State (FBS)

**Figure 13:**
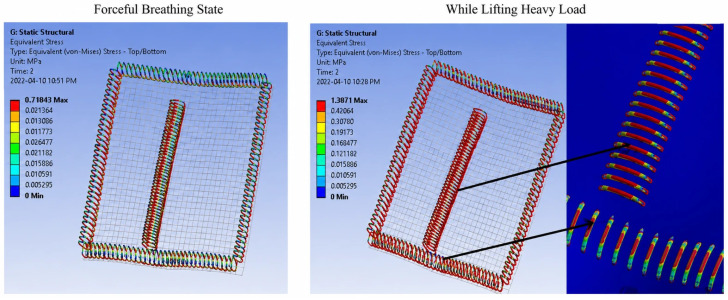
Equivalent stress distribution in the sutures during forceful breathing and heavy lifting.

**Figure 14:**
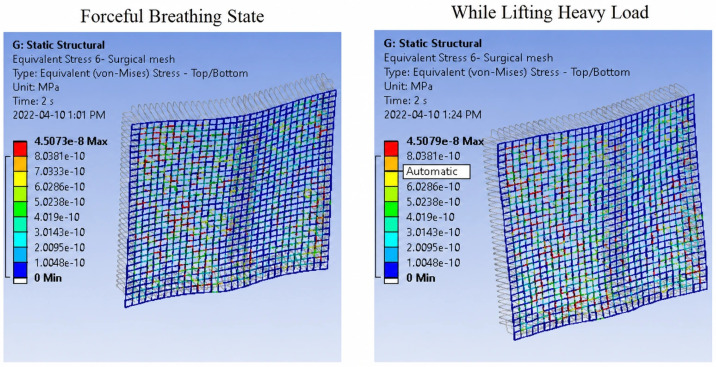
Finite Element Stress Analysis of the Surgical Mesh During Forceful Breathing and Heavy-Load Lifting.

**Figure 15:**
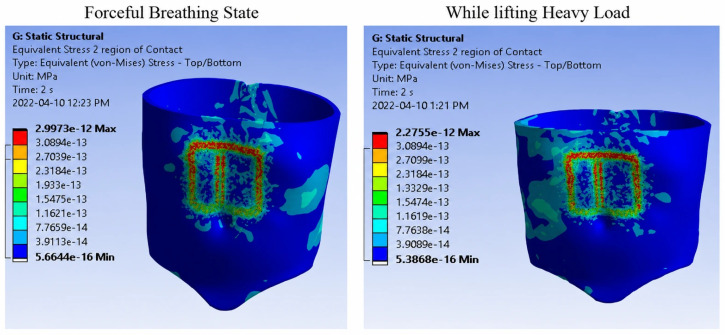
Contact stress distribution on the abdominal wall under forceful breathing and heavy lifting.

During the forceful breathing state, an average load of 38.25 N was applied to the model. The resulting stress contours (Figure 13, Figure 14 and Figure 15) demonstrated increased stress magnitudes compared to the regular breathing state. Stress concentrations remained primarily localized at the suture edges and at the suture–muscle contact regions. Notably, stresses at the suture edges were consistently higher than those observed in the surrounding contact areas, highlighting the susceptibility of sutures to mechanical overload during elevated physiological activity.

### 3.5. Stress Distribution During Heavy-Load Lifting

**Figure 16:**
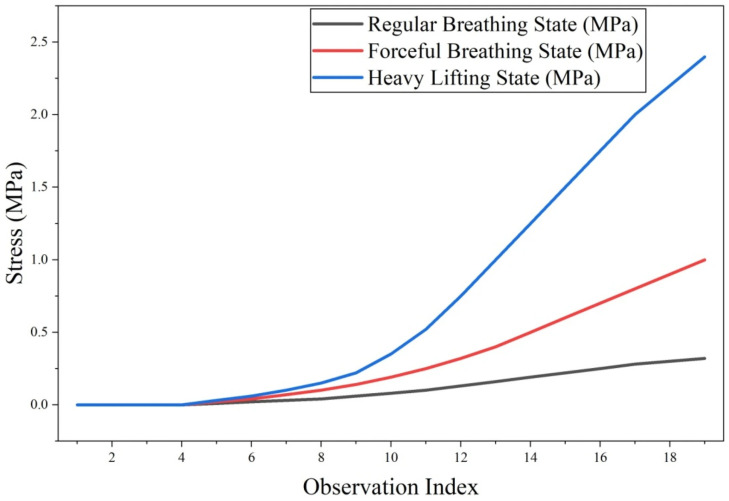
Variation in von Mises Stress Under Regular Breathing, Forceful Breathing, and Heavy Lifting.

The highest stress levels were observed during the heavy-load lifting state, where an average force of 74.3 N was applied to the model. The stress contours (Figure 16) showed pronounced stress concentrations at the sutures, contact interfaces, and, to a lesser extent, within the surgical mesh. These results indicate that lifting heavy loads substantially increases the mechanical demand on the repair system, thereby elevating the risk of suture failure and mesh displacement.

### 3.6. Deformation and Displacement Analysis

Under the regular breathing state, only minor deformation was observed, indicating that the repair system was able to maintain adequate structural stability under normal physiological loading conditions. As the loading increased to the forceful breathing state, the magnitude of displacement increased correspondingly, although the deformation remained localized within the vicinity of the repair site. The greatest displacement occurred during heavy-load lifting, reflecting the higher mechanical demand imposed on the abdominal wall–mesh–suture system.

The progressive increase in displacement with increasing loading conditions demonstrates that deformation behavior is strongly influenced by intra-abdominal pressure. Although the surgical mesh provided structural reinforcement, excessive loading resulted in larger deformations and increased stress concentrations around the fixation points. These findings suggest that excessive postoperative loading may compromise the mechanical stability of the repair and contribute to the development of recurrence.

### 3.7. Comparative Analysis of Stress Across Physiological States

A comparative assessment of stress magnitudes across the three physiological states demonstrated a clear increasing trend:

(1) Regular Breathing State<Forceful Breathing State<Heavy Load Lifting State

This progression confirms that activities involving higher intra-abdominal pressure generate significantly greater stress within the hernia repair system. The sutures consistently experienced the highest stress concentrations across all loading conditions, followed by the suture–muscle contact regions and the surgical mesh. These findings underscore the critical role of suture integrity in preventing hernia recurrence and highlight the importance of postoperative activity management to minimize excessive loading on repaired abdominal walls.

From a clinical perspective, the results suggest that optimization of suture spacing may play an important role in improving the mechanical stability of hernia repairs. Smaller suture spacing may promote more uniform load sharing among fixation points and reduce localized stress concentrations, thereby decreasing the likelihood of suture pull-through or rupture. Conversely, widely spaced sutures may result in higher stresses being transmitted to individual fixation points, increasing the risk of mechanical failure.

The present findings also emphasize the importance of the mesh fixation strategy. Alternative fixation configurations and improved load distribution mechanisms may reduce stress concentrations at the mesh–suture interface and enhance the long-term integrity of the repair construct. Appropriate mesh positioning and fixation patterns could therefore contribute to reducing recurrence rates and improving clinical outcomes.

Furthermore, the observed increase in stress under heavy-load lifting conditions highlights the importance of postoperative rehabilitation and activity management. Patients recovering from ventral incisional hernia repair should avoid excessive abdominal loading during the early stages of healing, as strenuous activities and heavy lifting may expose the sutures to stresses approaching their mechanical limits. Consequently, controlled rehabilitation protocols and a gradual return to physical activity may be essential for preserving repair integrity and minimizing the risk of recurrence.

### 3.8. Limitations

Several limitations of the present study should be acknowledged [25,26,27]. First, the abdominal wall was represented as a single equivalent isotropic hyperelastic layer rather than as four distinct muscle layers, namely the rectus abdominis, external oblique, internal oblique, and transversus abdominis. Although this simplification reduced computational complexity and facilitated convergence of the patient-specific finite element model, it may influence the magnitude and localization of stresses, particularly near the mesh–suture interface where stress concentrations are highest. Furthermore, the anisotropic behavior and fiber orientations of the individual muscle layers were not considered because such information could not be reliably obtained from the available CT images.

Second, the loading conditions employed in the simulations were simplified by assuming a uniformly distributed intra-abdominal pressure acting over the abdominal surface. In reality, intra-abdominal pressure varies spatially and temporally and may be influenced by patient posture, breathing patterns, and different physical activities. Consequently, the applied loading conditions represent an approximation of the physiological environment rather than an exact reproduction of in vivo conditions.

Third, the present study focused primarily on stress transfer mechanisms within the abdominal wall–mesh–suture system and did not consider time-dependent tissue remodeling, healing processes, or the viscoelastic effects of biological tissues. These factors may influence the long-term mechanical behavior of the repair and contribute to hernia recurrence.

Finally, experimental validation of the numerical predictions has not yet been performed. Although the simulation results are in agreement with clinical observations regarding the role of suture failure in recurrent hernias, further validation using physical phantoms, cadaveric specimens, and animal models is required to establish the reliability and predictive capability of the developed finite element framework. Future studies will focus on incorporating multilayer anisotropic constitutive models, dynamic loading conditions, and experimental validation to provide a more physiologically realistic representation of ventral incisional hernia repair.

## 4. Conclusions

This study investigated the mechanical behavior of a ventral incisional hernia repair system using a finite element analysis (FEA) model incorporating the abdominal wall, surgical mesh, and sutures under physiologically relevant loading conditions. The primary objective was to evaluate stress transfer within the repair system when intra-abdominal contents exert pressure on the weakened abdominal wall during activities such as breathing and heavy lifting. The developed FEA framework successfully captured the stress distribution within the system and provided insight into the mechanical factors contributing to hernia recurrence.

The results demonstrated that the highest stress concentrations consistently occurred at the sutures rather than within the mesh or the surrounding abdominal wall tissue. As the applied loading increased, particularly under conditions simulating heavy lifting, the stresses within the sutures progressively approached their yield strength. These findings indicate that suture failure is a critical mechanical trigger for the recurrence of ventral incisional hernias. Importantly, this numerical outcome aligns closely with empirical clinical observations reported by surgeons, who commonly identify suture rupture or pull-through as the primary failure mode in recurrent hernia cases, especially in patients exposed to high postoperative loads.

From a clinical perspective, the findings suggest that optimization of suture spacing and mesh fixation strategies may play an important role in reducing stress concentrations and improving the long-term stability of the repair. More uniform load distribution achieved through appropriate fixation patterns could potentially decrease the likelihood of suture overload and recurrence. Furthermore, the results emphasize the importance of postoperative rehabilitation and activity management. Avoidance of excessive abdominal loading during the early healing period, particularly during heavy lifting and strenuous activities, may help preserve repair integrity and improve long-term surgical outcomes. Therefore, the developed model has potential value as a predictive tool for surgical planning and postoperative care strategies.

The agreement between the FEA predictions and clinical experience strengthens the credibility of the developed model and highlights its usefulness for understanding the biomechanical mechanisms associated with ventral incisional hernia recurrence. Future research will extend this work in several directions. First, a more anatomically realistic abdominal wall model incorporating all four muscle layers with distinct anisotropic mechanical properties will be developed to better represent in vivo behavior. Second, a dynamic FEA framework will be introduced to capture large deformations and time-dependent loading associated with daily activities. Third, experimental validation using physical phantoms and animal models will be conducted to further assess the reliability and accuracy of the numerical predictions. Finally, ongoing work aims to develop an adaptive “smart” postoperative abdominal binder capable of real-time load modulation, offering personalized mechanical support and potentially reducing the risk of hernia recurrence.

## Figures and Tables

**Table 1:** Force ranges exerted on the abdominal wall under different physiological conditions (male participants) [24].

Participant No.	Regular Breathing—Expiration (N)	Forceful Breathing—Expiration (N)	Heavy Lifting (N)
1	13.7	33.3	51.2
2	11.2	51.8	40.7
3	11.2	33.3	77.3
4	16.3	30	64.5
5	15.2	28.6	83.4
6	15.4	53.1	78.2
7	17.7	30.9	56.9
8	21.6	38.2	82.7
9	25.1	44.4	85.9
10	13.1	38.9	122.2

## Data Availability

No new data were created or analyzed in this study. Data sharing is not applicable to this article.
